# Neonatal hemochromatosis with εγδβ-thalassemia: a case report and analysis of serum iron regulators

**DOI:** 10.1186/s12887-022-03706-3

**Published:** 2022-10-29

**Authors:** Mitsuru Tsuge, Aya Kodera, Hiromi Sumitomo, Tooru Araki, Ryuichi Yoshida, Kazuya Yasui, Hiroki Sato, Yosuke Washio, Kana Washio, Kenji Shigehara, Masato Yashiro, Takahito Yagi, Hirokazu Tsukahara

**Affiliations:** 1 Department of Pediatric Acute Disease, Okayama University Academic Field of Medicine, Dentistry, and Pharmaceutical Sciences, 2-5-1 Shikata-cho, Kita-ku, Okayama, 700-8558 Japan; 2 Department of Pediatrics, Fukuyama City Hospital, Fukuyama, Japan; 3 Department of Pediatrics, Matsuyama Red Cross Hospital, Matsuyama, Japan; 4 Department of Pediatrics, National Hospital Organization Fukuyama Medical Center, Fukuyama, Japan; 5 Department of Gastroenterological Surgery, Okayama University Hospital, Okayama, Japan; 6 Department of Pediatrics, Okayama University Hospital, Okayama, Japan; 7 Department of Pediatrics, Okayama University Academic Field of Medicine, Dentistry, and Pharmaceutical Sciences, Okayama, Japan

**Keywords:** Neonatal hemochromatosis, Thalassemia, Liver transplantation, Gestational alloimmune liver disease, Case report, Hepcidin, Ineffective erythropoiesis, Growth differentiation factor-15

## Abstract

**Background:**

Neonatal hemochromatosis causes acute liver failure during the neonatal period, mostly due to gestational alloimmune liver disease (GALD). Thalassemia causes hemolytic anemia and ineffective erythropoiesis due to mutations in the globin gene. Although neonatal hemochromatosis and thalassemia have completely different causes, the coexistence of these diseases can synergistically exacerbate iron overload. We report that a newborn with εγδβ-thalassemia developed neonatal hemochromatosis, which did not respond to iron chelators and rapidly worsened, requiring living-donor liver transplantation.

**Case presentation:**

A 1-day-old Japanese boy with hemolytic anemia and targeted red blood cells was diagnosed with εγδβ-thalassemia by genetic testing, and required frequent red blood cell transfusions. At 2 months after birth, exacerbation of jaundice, grayish-white stool, and high serum ferritin levels were observed, and liver biopsy showed iron deposition in hepatocytes and Kupffer cells. Magnetic resonance imaging scans showed findings suggestive of iron deposits in the liver, spleen, pancreas, and bone marrow. The total amount of red blood cell transfusions administered did not meet the criteria for post-transfusion iron overload. Administration of an iron-chelating agent was initiated, but iron overload rapidly progressed to liver failure without improvement in jaundice and liver damage. He underwent living-donor liver transplantation from his mother, after which iron overload disappeared, and no recurrence of iron overload was observed. Immunohistochemical staining for C5b-9 in the liver was positive. Serum hepcidin levels were low and serum growth differentiation factor-15 levels were high prior to living-donor liver transplantation.

**Conclusions:**

We reported that an infant with εγδβ-thalassemia developed NH due to GALD, and that coexistence of ineffective erythropoiesis in addition to erythrocyte transfusions may have exacerbated iron overload. Low serum hepcidin levels, in this case, might have been caused by decreased hepcidin production arising from fetal liver damage due to neonatal hemochromatosis and increased hepcidin-inhibiting hematopoietic mediators due to the ineffective hematopoiesis observed in thalassemia.

**Supplementary Information:**

The online version contains supplementary material available at 10.1186/s12887-022-03706-3.

## Background

Neonatal hemochromatosis (NH) is a rare disease characterized by severe systemic iron deposition that develops in cases of acute liver failure at birth. The prognosis for NH is poor without treatment, often leading to fetal loss or neonatal death. Liver transplantation is considered for severe cirrhosis due to NH [1].

Thalassemia is a hereditary hemolytic anemia caused by mutations in globin genes that make up hemoglobin. Severe thalassemia may present with iron overload due to frequent blood transfusions. Εγδβ-thalassemia is a rare type of thalassemia that lacks expression of all non-α-globin genes and causes severe hemolytic anemia during fetal and neonatal periods but becomes mild in the first few months of life [2].

There are no reports of cases with coexistent NH and thalassemia. Although the causes of these two illnesses are completely different, both NH and thalassemia are known to be associated with iron overload. It has not been clarified whether the coexistence of these diseases may further exacerbate systemic iron overload. Moreover, there are no reports of changes in serum iron metabolism regulators in such cases.

We present a case of a Japanese boy with εγδβ-thalassemia who developed NH after birth; despite the administration of iron chelators, iron overload progressed rapidly, requiring living-donor liver transplantation.

## Case presentation

**Fig. 1:**
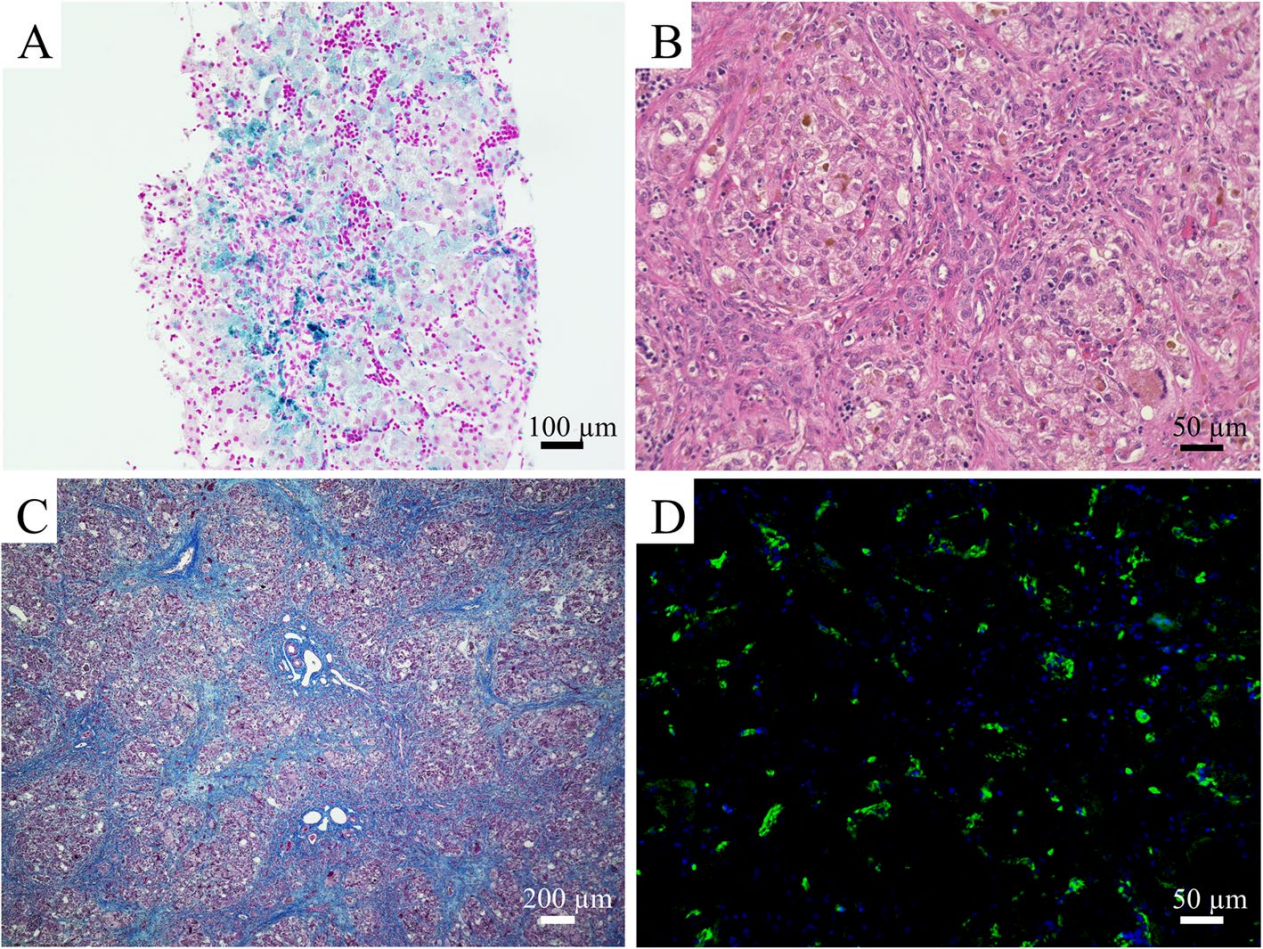
Histopathological examination of the liver tissue. Histopathological examination of the liver tissues identified (**A**) Iron deposition in hepatocytes and Kupffer cells in the biopsied liver tissue (Berlin blue, × 100) (**B**) Multinucleated giant hepatocytes, ballooning hepatocytes, intrahepatic cholestasis, bile canaliculus plugs, and mononuclear cell infiltration (hematoxylin and eosin stain [H&E], ×200) (**C**) Interlobular fibrosis (Masson Trichrome, ×40) (**D**) C5b-9 positive stained hepatocytes (green) with nuclear staining (blue) (immunohistochemistry, × 200)

**Fig. 2:**
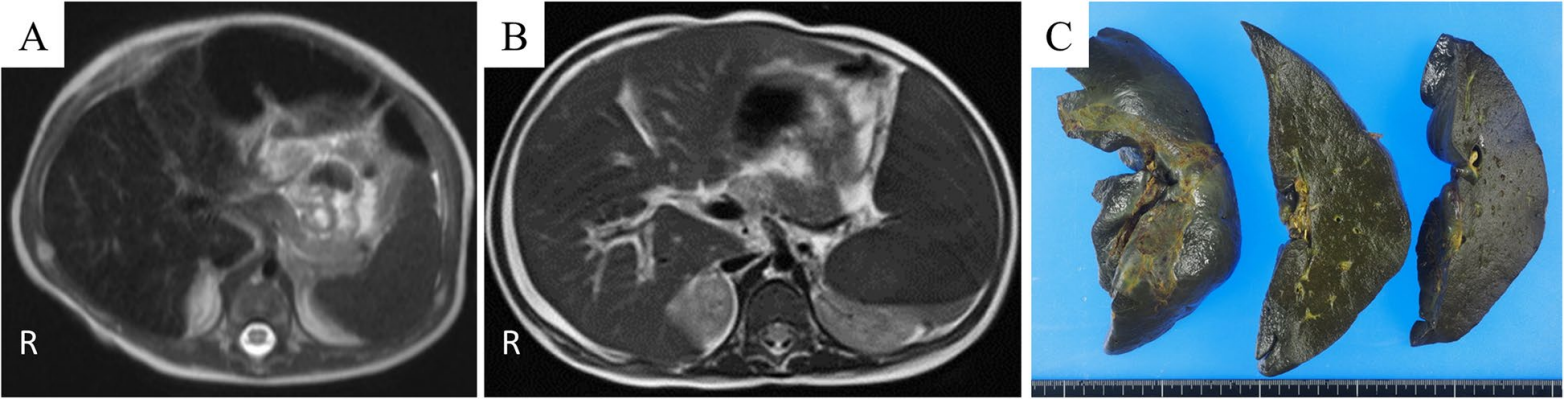
Magnetic resonance imaging and photograph of the liver. Coronal T2 weighted magnetic resonance images of the abdomen at 3 months of age (**A**) and 5 months of age (**B**), showing hepatosplenomegaly and diffuse low-intensity of liver, pancreas, spleen and spinal bone marrow due to iron deposition. (**C**) Gross photograph of resected liver, demonstrating a dark green cut surface

A one-day-old Japanese boy was admitted to a nearby medical institution for jaundice, and his blood tests showed an elevated indirect bilirubin level and microcytic hypochromic anemia. Serum haptoglobin levels were low and target erythrocytes were found in peripheral blood smears, suggesting the possibility of hemolytic anemia due to thalassemia. His parents are Japanese and not consanguineous. He had no family history of liver or blood disease. There was no abnormality in the perinatal period, and he was born at term. No abnormalities were found in screening tests for congenital metabolic disorders. He needed a red blood cell transfusion every 2 weeks after birth for progressive anemia. Grayish-white stools appeared with persistent jaundice, and his blood tests at 67 days of age showed an increase in serum bilirubin levels (total bilirubin 12.7 mg/dL, direct bilirubin 7.6 mg/dL), transaminases (aspartate aminotransferase [AST], 198 IU/l; alanine aminotransferase [ALT], 190 IU/L), total bile acid (34.8 μmol/L), and alpha-fetoprotein (174,649 ng/mL). No increases in γ-glutamyl transpeptidase, prothrombin time-international normalized ratio (PT-INR), or ammonia were observed. Increased serum levels of iron (139 mg/dL), ferritin (2948 ng/mL), and transferrin saturation (91%) were observed. Levels of antinuclear antibody, blood glucose, thyroid hormone, and α1 antitrypsin were all within the normal range. There was no serological evidence of hepatitis virus (A, B, C, and E), cytomegalovirus, Epstein-Barr virus, herpes simplex virus, mumps, measles, rubella, varicella-zoster virus, parvovirus B19, *treponema pallidum*, or toxoplasma. All culture tests were negative. Amino acid analysis in blood did not reveal any findings suggestive of congenital amino acid metabolism disorders. Although abdominal ultrasonography showed no abnormalities in the bile ducts, bile duct scintigraphy and duodenal fluid examination showed poor bile outflow. Ascites, a slightly hard liver, and dilated gallbladder filled with yellow bile were observed during laparotomy. All biliary tracts of the common bile duct, common hepatic duct, and intrahepatic bile duct were visualized on cholangiography; therefore, biliary atresia was excluded. Liver biopsy showed changes in hepatocellular giant cells, bile canalicular plugs, and mononuclear cell infiltration. Berlin blue staining revealed that iron was deposited in hepatocytes and Kupffer cells (Fig. 1A). Low-intensity signals on T2-weighted magnetic resonance (MR) images were observed in the liver, pancreas, spleen, and bone marrow (Fig. 2A). Analysis of 61 genes responsible for hereditary disorders associated with neonatal and infantile cholestasis did not reveal any mutations (Additional file 1). A genetic test for thalassemia confirmed the diagnosis of εγδβ-thalassemia.

He was diagnosed with NH based on neonatal-onset cholestatic hepatitis, high transferrin saturation, high serum levels of ferritin and α-fetoprotein, iron deposits in liver tissue, and MR images suggesting iron deposition in organs other than the liver. The total amount of red blood cell transfusion was approximately 40 mL/kg at the time of initiating the iron-chelating agent. An intravenous infusion of deferoxamine (60 mg/kg/day) was started at 86 days of age in addition to oral administration of fat-soluble vitamins, medium-chain triglycerides, glycyrrhizin, ursodeoxycholic acid, phenobarbital, and lactulose. Intravenous deferoxamine was changed to oral administration of deferasirox (12 mg/kg/day) at 105 days of age. Some partial blood exchange transfusions were performed, but intravenous immunoglobulin was not administered. Subsequently, no decrease in serum levels of ferritin, bilirubin, or transaminase was observed; therefore, he was transferred to our hospital at 5 months (160 days) of age.

On admission to our hospital, his length was 66.0 cm (− 0.1 SD) and weight 7.3 kg (− 0.5 SD) with no delay in physical growth or mental development. His urine was dark brown, and his stool was grayish-white. His skin color was brownish bronze, and his bulbar conjunctiva was markedly yellowed. He was a little drowsy but had no neurological abnormalities. He had abdominal distension and hepatosplenomegaly. His blood tests showed increased serum transaminase levels (AST 716 IU/L and ALT 338 IU/L), increased serum bilirubin levels (total bilirubin 45.9 mg/dL, direct bilirubin 33.6 mg/dL), increased serum ferritin level (4864 ng/mL), and increased iron saturation level (73%). Elevated levels of serum hyaluronic acid (459.3 ng/mL), a liver fibrosis marker, was observed. No abnormalities were found in blood ammonia concentration or electroencephalogram. Computed tomography of the abdomen showed hepatosplenomegaly, periportal collar signs around the portal vein, and ascites. No improvement was observed in the low-intensity signals of the liver, pancreas, spleen, or bone marrow on T2-enhanced MR images (Fig. 2B). No mutations in genes associated with hereditary hemochromatosis (*HFE*, *HJV*, *HAMP*, *TFR2*, and *SLC40A1*) were found. After admission, serum ferritin levels gradually decreased, but serum levels of bilirubin and transaminase gradually increased. In addition, thrombocytopenia (41,000 /μL), decreased serum albumin levels (2.1 g/dL), and increased PT-INR (1.19) manifested. The Child-Pugh score was 8 points, and the Pediatric End-stage Liver Disease (PELD) score was 19 points. He was diagnosed with irreversible liver failure, and his parents agreed with the necessity of living-donor liver transplantation after receiving sufficient explanation. He underwent living-donor liver transplantation with an enlarged left lateral segment graft from his mother at 6 months of age (202 days of the age). The graft weighed 310 g, and the graft-to-recipient weight ratio was 3.88. Immunosuppressive therapy consisted of steroids and tacrolimus. His resected liver was macroscopically dark green, suggesting strong iron deposition and cholestasis (Fig. 2C). Pathological examination of his excised liver revealed multinucleated giant hepatocytes, ballooning hepatocytes, intrahepatic cholestasis, bile canaliculus plugs, mononuclear cell infiltration, and interlobular fibrosis (Fig. 1B and C). Immunohistochemical analysis of the liver was positive for C5b-9 using rabbit polyclonal antibody (bs2673R; Bioss Antibodies, Woburn, MA, USA) (Fig. 1D). After liver transplantation, serum bilirubin, transaminases, and ferritin levels gradually decreased, and splenomegaly improved. Deferasirox was discontinued on the 56th day after surgery, and no re-elevation of the ferritin level was observed thereafter. At the same time, the progression of anemia associated with thalassemia slowed down, the frequency of red blood cell transfusions decreased, and he did not need blood transfusions after being discharged 2 months after transplantation. Currently, we are continuing immunosuppressive therapy with tacrolimus and steroids and following up on liver function and his growth and development. No recurrence of NH was observed from the time of living-donor liver transplantation to 1 year 9 months of age. His blood tests showed mildly elevated serum transaminase levels (AST 41 IU/L and ALT 84 IU/L), normal serum bilirubin levels (total bilirubin 1.38 mg/dL, direct bilirubin 0.38 mg/dL), normal serum ferritin level (56.3 ng/mL), and normal hemoglobin level (11.1 g/dL) at 1 year 9 months of age. In addition, we have continued assessing organ function such as the thyroid gland, pancreas, and heart in consideration of systemic iron deposits caused by NH, but no abnormal findings have been found. The definitive treatment for thalassemia is bone marrow transplantation, but εγδβ-thalassemia is known to be mild in infancy. Currently, his blood test still shows low haptoglobin levels, but he has shown no progression of anemia and is not dependent on blood transfusions. Considering the risk of bone marrow transplantation, we will continue to follow up on his anemia without bone marrow transplantation, with the consent of his parents.

Serum hepcidin-25 and growth differentiation factor (GDF)-15 levels were measured to investigate the changes in iron metabolism regulators in this case (Table 1). The level of serum hepcidin-25, a negative regulator of iron utilization in the intestinal or reticular endothelial system, was not increased at the time of transfer and gradually declined to below the normal range. Conversely, the serum level of GDF-15, which can inhibit hepcidin-25, was higher than the normal range at transfer and increased thereafter.

**Table 1:** Changes in serum levels of parameters of cholestatic liver damage, iron homeostasis and erythropoiesis

Days after birth	9	28	67	86	118	163	170	177	184	191	202
Hgb (g/dL) (NR: 13.6–18.3)	6.8	6.7	6.3	7.9	6.3	8.9	7.3	7.7	8.7	6.0	7.0
Reticulocyte count (%) (NR: 0.2–2.0)	ND	ND	ND	9.6	10.4	4.3	ND	ND	3.6	ND	ND
T-Bil (mg/dL) (NR: 0.3–1.2)	18.7	15.1	12.7	14.1	38.3	45.9	44.5	45.5	46.1	45.5	45.6
D-Bil (mg/dL) (NR: < 0.4)	1.2	2.2	7.6	9.7	26.2	33.6	32.3	34.1	34.6	34.4	32.7
AST (IU/L) (NR: 10–40)	13	25	198	365	446	716	730	777	756	956	1381
ALT (IU/L) (NR: 5–45)	10	24	190	302	275	338	322	298	262	294	320
Iron (μg/dL) (NR: 60–210)	134	147	139	158	415	479	569	581	557	476	414
Ferritin (ng/mL) (NR: 21–282)	596	795	2948	6412	1829	4864	3160	2422	2935	1784	1430
TIBC (μg/dL) (NR: 250–410)	199	178	152	140	602	652	721	711	680	642	554
Iron saturation (%) (NR: 20–40)	67	83	91	92	69	73	79	82	82	74	75
Hepcidin-25 (ng/mL) (NR: 0.8–14.8)	ND	ND	ND	ND	ND	5.1	1.2	0.5	0.8	0.1	0.3
GDF-15 (pg/mL) (NR: 337–1060)	ND	ND	ND	ND	ND	1123	1110	1358	1592	1978	2168

Abbreviations: *Hgb* Hemoglobin, *NR* Normal range, *T-Bil* Total bilirubin, *D-Bil* Direct bilirubin, *AST* Asparate aminotransferase, *ALT* Alanine aminotransferase, *TIBC* Total iron binding capacity, *GDF-15* Growth differentiation factor 15, *ND* No data

## Discussion and conclusions

Iron overload is a disease in which iron accumulates excessively due to abnormal iron metabolism, resulting in damage to various organs such as the liver, heart, pancreas, skin, thyroid gland, gonads, and salivary glands [3]. It is classified as “primary iron overload” caused by gene mutations related to iron metabolism and “secondary iron overload” caused by other causes. Hereditary hemochromatosis (HH), which accounts for the majority of the primary iron overload. Although HH caused by mutations in the hemojuvelin gene is known to occur in newborns and children [4], the frequency of HH in Japan is much less common than in Western countries [5]. This patient had no family history of hemochromatosis, and no mutations were found in several HH-related genes in this patient, suggesting a low possibility of HH. On the other hand, the causes of secondary iron overload include long-term frequent red blood cell transfusion, anemia with ineffective erythropoiesis, long-term administration of iron medication, chronic liver disease, and NH. In Japan, the frequency of post-transfusion iron overload is particularly high [5].

Most NH cases are thought to be gestational alloimmune liver disease (GALD) caused by the transplacental transfer of maternal immunoglobulin G (IgG) antibodies that react with fetal hepatocytes [6]. This complement-mediated immune response damages fetal hepatocytes and causes subsequent iron deposition [7]. In this case, immunohistochemical analysis of the liver was positive for C5b-9, indicating that antibody-mediated liver injury as GALD was the cause of NH in this case. The time of onset depends on the time of liver damage and varies from 18 weeks gestation to 3 months of age. In this case, the serum ferritin level was increased gradually after birth and developed liver damage with cholestasis at 2 months of age, suggesting that this case may have been late-onset NH. However, the causes of NH include not only GALD but also mitochondrial DNA depletion syndrome [8], bile acid synthesis deficiency, tyrosinemia, and hemophagocytic lymphohistiocytosis. No elevated C-reactive protein, hyperlactatemia, increased urinary succinylacetone levels, or abnormal bile acid metabolism were observed in this case, but we were unable to completely rule out all causes of NH other than GALD.

For treating iron overload, iron chelators have been reported to reduce serum ferritin levels, reduce organ damage, and prolong life [9–11]. However, in this case, despite commencing administration of an iron-chelating agent, the increase in serum ferritin levels could not be fully suppressed, and liver damage progressed. Fortunately, no recurrence of iron overload was observed after living-donor liver transplantation from the mother. This may be due to the fact that transplacentally transferred mother-derived IgG antibodies do not affect the transplanted mother’s liver. The efficacy of high-dose intravenous immunoglobulin (IVIG) therapy and exchange transfusion in the early stages of the onset of GALD-induced NH has been reported, and a significant improvement in survival has been observed [12]. Unfortunately, partial blood exchange transfusion was performed in this case, but IVIG therapy was not considered and was not performed in the early stages of the disease. If these treatments were administered early in this case, it may have been possible to suppress the immune response of GALD, improve liver damage earlier, and avoid the requirement for living-donor liver transplantation.

The causes of NH and thalassemia are completely different, but both are diseases that can be associated with secondary iron overload. The coexistence of thalassemia may have promoted iron deposition in NH. Severe thalassemia requires long-term frequent red blood cell transfusions, which can lead to post-transfusion iron overload. Since the human body does not have a system that actively excretes excess iron, iron derived from transfused red blood cells accumulates in the body [13]. Since εγδβ-thalassemia shows severe anemia during the neonatal period, red blood cell transfusions may have worsened the iron overload due to NH in this case. However, the total dose of red blood cell transfusion before the administration of iron-chelating agent was 40 mL/kg, which did not meet the Japanese diagnostic criteria for post-transfusion iron overload (50 mL/kg) [14], suggesting that iron overload due to other factors may have compounded the iron overload in NH.

The living body has a strict control mechanism that maintains iron homeostasis, which is regulated mainly by the absorption of iron from the digestive tract and the excretion of iron from the liver and reticuloendothelial cells [15, 16]. Serum iron is supplied via ferroportin (FPN), which is an iron efflux transporter [17]. Most iron is reused from waste erythrocytes captured by reticuloendothelial macrophages and released into the blood via FPN. In addition, iron from the diet is mainly absorbed from the intestinal epithelial cells in the duodenum and proximal jejunum and is released into the blood vessels via FPN. Ferroportin expression is regulated by hepcidin, an iron metabolism regulator. Hepcidin is produced by the liver, and hepcidin-25 is primarily involved in the regulation of iron metabolism. When hepcidin binds to FPN, FPN is broken down and iron excretion is reduced, resulting in a decrease in serum iron levels [18]. Hepcidin secretion is increased in response to iron overload, inflammation, and bone marrow failure and decreased in response to iron deficiency, hypoxia, anemia, and increased hematopoiesis [19]. In patients with HH, serum hepcidin is usually undetectable, despite iron overload, due to gene mutations involved in hepcidin expression. However, in this case, serum hepcidin level at the time of transportation to our hospital was within the normal range and subsequently declined to low levels.

The low serum hepcidin levels observed in this case may also be due to the liver damage caused by GALD in NH. Although few reports have investigated serum hepcidin levels in patients with NH, it has been reported that hepcidin expression in the liver tissue of most infants with NH was low, being about 20% of that in the liver tissue of normal neonates [20]. The supply of iron from the mother to the fetus is via placenta FPN, and the amount of iron that passes through the placenta is tightly controlled by hepcidin produced in the fetal liver. When hepcidin production is reduced due to fetal liver damage caused by GALD, placental FPN inhibition is thought to decrease and excess iron flows transplacentally and accumulates in the fetal parenchymal organs [21].

In addition, another possible cause of low serum hepcidin levels in this case was ineffective erythropoiesis due to thalassemia [22]. Enhanced erythroid hematopoiesis excessively suppresses hepcidin production. Thalassemia patients who are not dependent on blood transfusions can also develop iron overload and low serum hepcidin levels have been reported in patients with thalassemia [23]. Ineffective hematopoiesis in thalassemia promotes erythroblast formation in the bone marrow and promotes the secretion of GDF-15 or erythroferrone from erythroblast progenitor cells [22, 24]. These factors suppress hepcidin production in the liver, increase iron absorption from the gastrointestinal tract and iron supply from reticuloendothelial cells, and promote an increase in iron supply to the bone marrow. Serum GDF-15 and serum erythroferrone levels in thalassemia patients have been reported to be high [25, 26], and in this case, serum GDF-15 levels before liver transplantation were high. Congenital iron-loading anemia with ineffective erythropoiesis includes not only thalassemia but also congenital erythropoietic anemia, hereditary sideroblastic anemia, and erythrocyte pyruvate kinase deficiency. The serum level of GDF-15 was reported to be high in these anemias [27–29], and the coexistence with NH might exacerbate iron overload and make treatment with iron chelators difficult [30]. Future studies involving a larger number of such cases using iron metabolism-related mediators are needed.

Herein, we reported the case of an infant with εγδβ-thalassemia and NH. In addition to liver damage caused by GALD, iron load due to blood transfusion and suppression of hepcidin production due to ineffective erythropoiesis may have further exacerbated iron overload in the NH.

## Supplementary Information

**Additional file 1: Table S1.** Sixty-one genes in the analysis for the hereditary disorders associated with neonatal and infancy cholestasis.

## Data Availability

The original contributions presented in the study are included in the article/supplementary material, further inquiries can be directed to the corresponding author.

## Abbreviations

NH: Neonatal hemochromatosis
AST: Aspartate aminotransferase
ALT: Alanine aminotransferase
PT-INR: Prothrombin time-international normalized ratio
MR: Magnetic resonance
PELD: Pediatric end-stage liver disease
GDF-15: Growth differentiation factor-15
GALD: Gestational alloimmune liver disease
IVIG: Intravenous immunoglobulin
IgG: Immunoglobulin G
FPN: Ferroportin
