# Supplementary material for: Neonatal hemochromatosis with εγδβ-thalassemia: a case report and analysis of serum iron regulators
Source: BMC Pediatr. 2022 Oct 29;22:622. doi: 10.1186/s12887-022-03706-3 (PMC9617355; doi:10.1186/s12887-022-03706-3)
Supplement: Supplementary file 1 — Additional file 1: Table S1. Sixty-one genes in the analysis for the hereditary disorders associated with neonatal and infancy cholestasis. [file 12887_2022_3706_MOESM1_ESM.docx]

**Table S1.** Sixty-one genes in the analysis for the hereditary disorders associated with neonatal and infancy cholestasis

| **Gene name** | **Disease** | **Gene name** | **Disease** | **Gene name** | **Disease** |
| --- | --- | --- | --- | --- | --- |
| *JAG1, NOTCH2* | Alagille syndrome | *CFTR* | Cystic fibrosis | *NPHP1, NPHP3, NPHP4, INVS* | Nephronophthisis |
| *SEAPINA1* | Alpha-1-antitrypsin deficiency | *ABCC2* | Dubin-Johnson syndrome | *NPC1, NPC2* | Niemann-Pick disease, type C |
| *AMACR* | Alpha-methylacyl-CoA racemase deficiency | *BAAT* | Familial hypercholanemia 2 | *PKHD1, PKD2, SEC63, PRKCSH* | Polycystic kidney and hepatic disease |
| *VPS33B, VIPAS39* | Arthrogryposis-Renal dysfunction-Cholestasis syndrome | *GALT* | Galactosemia | *ATP8B1, ABCB11, ABCB4, TJP2, NR1H4* | Progressive Familial Intrahepatic Cholestasis |
| *SLC27A5* | Bile acid amidation defect | *TRMU* | Infantile liver failure | *SLCO1B1, SLCO1B3* | Rotor Syndrome |
| *HSD3B7, AKR1D1, CYP7B1* | Bile Acid Synthetic defect | *CC2D2A, MKS1* | Joubert syndrome/Meckel syndrome | *DHCR7* | Smith-Lemli-Opitz syndrome |
| *CYP7A1* | Bile acid synthesis genes | *LIPA* | Lysosomal acid lipase deficiency | *FAH* | Tyrosinemia 1 |
| *HNF1B* | Bile duct development genes | *DGUOK, MPV17, BCL1L, POLG* | Mitochondrial disorder | *ATP7B* | Wilson disease |
| *CYP27A1* | Cerebrotendinous xanthomatosis | *SLC25A13* | Neonatal intrahepatic Caused by Citrin Deficiency | *PEX1, PEX2, PEX3, PEX5, PEX6, PEX10, PEX12, PEX13, PEX14, PEX16, PEX19, PEX26* | Zellweger spectrum disorder |
| *UGT1A1* | Crigler Najjar/Gilbert syndrome | *CLDN1* | Neonatal sclerosing cholangitis | *ATP11C* | Coding aminophopholipid flippase |
